# Your Brain on the Movies: A Computational Approach for Predicting Box-office Performance from Viewer’s Brain Responses to Movie Trailers

**DOI:** 10.3389/fninf.2017.00072

**Published:** 2017-12-19

**Authors:** Christoforos Christoforou, Timothy C. Papadopoulos, Fofi Constantinidou, Maria Theodorou

**Affiliations:** ^1^Division of Computer Science, Mathematics and Science, St. John’s University, New York, NY, United States; ^2^Division of Research and Development, R.K.I Leaders Ltd., Larnaca, Cyprus; ^3^Center for Applied Neuroscience, University of Cyprus, Nicosia, Cyprus; ^4^Department of Psychology, University of Cyprus, Nicosia, Cyprus

**Keywords:** EEG, eye-tracking, neuro-cinematics, neuro-marketing, film test screening, pilot test screening

## Abstract

The ability to anticipate the population-wide response of a target audience to a new movie or TV series, before its release, is critical to the film industry. Equally important is the ability to understand the underlying factors that drive or characterize viewer’s decision to watch a movie. Traditional approaches (which involve pilot test-screenings, questionnaires, and focus groups) have reached a plateau in their ability to predict the population-wide responses to new movies. In this study, we develop a novel computational approach for extracting neurophysiological electroencephalography (EEG) and eye-gaze based metrics to predict the population-wide behavior of movie goers. We further, explore the connection of the derived metrics to the underlying cognitive processes that might drive moviegoers’ decision to watch a movie. Towards that, we recorded neural activity—through the use of EEG—and eye-gaze activity from a group of naive individuals while watching movie trailers of pre-selected movies for which the population-wide preference is captured by the movie’s market performance (i.e., box-office ticket sales in the US). Our findings show that the neural based metrics, derived using the proposed methodology, carry predictive information about the broader audience decisions to watch a movie, above and beyond traditional methods. In particular, neural metrics are shown to predict up to 72% of the variance of the films’ performance at their premiere and up to 67% of the variance at following weekends; which corresponds to a 23-fold increase in prediction accuracy compared to current neurophysiological or traditional methods. We discuss our findings in the context of existing literature and hypothesize on the possible connection of the derived neurophysiological metrics to cognitive states of focused attention, the encoding of long-term memory, and the synchronization of different components of the brain’s rewards network. Beyond the practical implication in predicting and understanding the behavior of moviegoers, the proposed approach can facilitate the use of video stimuli in neuroscience research; such as the study of individual differences in attention-deficit disorders, and the study of desensitization to media violence.

## Introduction

Anticipating the behavior of large audiences to video stimuli, such as movies, movie trailers and TV series, is critical for the film industry. Movie trailers serve as a primary marketing tool to promote a movie, and often capture the core characteristic of the movie and motivate its storyline. Thus, evaluating the responses of moviegoers to movie trailers can provide a convenient means of predicting an audience response to the actual movie and hence to anticipate its potential commercial success, before its release. Traditionally, film testing relies on measurements of consumers’ stated preferences, obtained via questionnaires, and focus groups conducted during a test screening of the movie or the movie trailer (Eastman and Ferguson, 2012). However, with more than 75% of new movie releases earning a net loss during their run in theaters (Boksem and Smidts, 2015), there is a need for new methods for predicting moviegoer’s behavior.

Recently, there has been a growing interest in using neural and biometric measures to identify metrics that predict the performance and effectiveness of video stimuli. These methods have been employed in a diverse set of applications, including investigating cross-culture (Vecchiato et al., 2014a) and gender (Vecchiato et al., 2014b) differences during the observation of video advertisements, analyzing movies to inform cognitive film theory (Smith, 2013), measuring engagement levels during video viewing (Dmochowski et al., 2012), predicting viewership of TV series (Dmochowski et al., 2014), predicting video advertising appreciation potential (Christoforou et al., 2015), and studying affective processing in individuals with high callous-unemotional traits (Fanti et al., 2016), among others. As movie trailers are a type of video stimuli, neuroscience metrics obtained while people are observing movie trailers might provide an alternative framework for predicting the overall performance of a film.

A testing framework based on neuroscience metrics can have several advantages over traditional methods. First, the noise variance of neuroscience metrics is thought to be smaller^1^ than data obtained through traditional measures (Boksem and Smidts, 2015). Thus, these metrics could provide more accurate insights with smaller sample sizes, making them potentially cheaper, faster to implement, and likely to provide more accurate predictions. Second, it is thought that neuroscience measurements have the potential to provide additional information that is not obtainable through conventional methods (i.e., questionnaires and focus groups). The rationale of this assertion relies on the assumptions that people cannot fully articulate their preferences when asked to express them explicitly and that consumers’ brain contains hidden information about their actual preferences. Also, stated preferences can be thought of as being the result of the underlying subconscious response of the brain “contaminated” by conscious cognitive control processes and individual biases. In this context, neuronal measures might be thought of as providing the direct, raw and unfiltered impression of viewers to video stimuli. Thus, neural measures might obtain information that better reflects viewers’ responses to stimuli, uncontaminated by personal biases. Finally, neuroscience measures may provide second-by-second responses to video stimuli, yielding information processing details that are not available through conventional methods. With these potential advantages in mind, this study proposes a novel approach for extracting meaningful neurophysiology and eye-gaze based metrics to predict the population-wide behavior of moviegoers.

To date, the most common methods used to measure preferences through neural activity have been electroencephalography (EEG) and functional magnetic resonance imaging (fMRI). For example, neural activity in response to advertisements, measured in the ventromedial Prefrontal Cortex (vmPFC) via fMRI, has been shown to be predictive of the population-wide success of commercials (Berns and Moore, 2012; Falk et al., 2012). Moreover, some methods have been proposed to extract informative metrics from neuronal activity captured in EEG measures. Such methods typically rely on obtaining spatial or temporal projections of the raw or pre-processed EEG measures to isolate neuronal activity relevant to a task (Dyrholm et al., 2007; Christoforou et al., 2008, 2013). Resulting metrics have been used in different applications such as Brain-computer interfaces (Blankertz et al., 2008), Robotic-telepresence (Christoforou et al., 2010) and maximization of throughput in performance tasks (Parra et al., 2008). In the context of video stimuli evaluation, EEG-based neuronal activity has been used to characterize the effects of video advertising on consumers. For example, Vecchiato et al. (2011) investigated the changes of EEG frontal asymmetry in the alpha and theta bands, during the observation of pleasant and unpleasant videos. Their analysis showed that an asymmetrical increase in the alpha and theta bands was negatively correlated with the degree of pleasances perceived by the participant. Similarly, Kong et al. (2012) proposed metrics which rely on the overall power in the theta band in EEG signals, as an index of memorization, and as an index of attention. In subsequent work, Kong et al. (2012) proposed the impression index, which combines both the memorization and attention indices. The authors suggested that the aggregate index tracks variations in cerebral activity, measured while subjects are observing video advertisements, could help judge whether scenes in the video advertising are impressive or not.

Recent efforts aim to identify measures of neuronal activity that carry predictive information regarding the performance of video stimuli. For example, Dmochowski et al. (2014) proposed the use of inter-subject correlation (ISC) to extract EEG components that are maximally correlated across participants during video viewing. The extracted ISC components are then used to define a metric that carries predictive information about viewership size and tweet frequency rates during a television broadcast. The same parameter calculated during observation of video advertisements could provide predictive information about the post-air preference rating of each advertisement. In another study, Boksem and Smidts (2015) investigated whether neural measures carry predictive information about the commercial success of movies, above and beyond information obtained through traditional, stated preference measures. In particular, they explored the overall power of high-frequency components (in beta and gamma bands) of EEG measurements obtained during the observation of a movie trailer as predictors of the box-office success of the film. Their results indicated that overall beta activity in EEG could provide predictive information about individual preferences, while overall gamma activity could carry predictive information about the population-wide success of the movie. The authors recognized that in both instances the predictive power of the neuronal measures was small (explained variance <2%), despite being statistically significant.

Various metrics which rely on eye-tracking measurements during video viewing have also been proposed as indicators of video content performance. Typically, these metrics rely on calculating a measure of consistency of eye-movements across different observers. Suggested metrics include: clustering-based methods (Goldstein et al., 2007), which measures the percentage of fixations falling within a cluster; string editing methods (Clauss et al., 2004), where gaze paths are encoded in string representation; attentional synchrony (Smith and Mital, 2013) and information theoretic metrics (Rajashekar et al., 2004); and other methods such as Dorr et al. (2010). A fundamental problem with such methods is that there is no direct (known) mapping between the eye-position and its perceptual consequences, and more importantly, none of these metrics has been shown to carry predictive information of the population-wide audience preferences (Dorr et al., 2010). More recently, Christoforou et al. (2015) proposed the eye-gaze divergence index; a metric that relies on the dispersion of eye-gaze points during video viewing across participants, and that has been shown to carry predictive information on the likability of narrative videos, in particular, video commercials.

In this study, we introduce a new approach for extracting neural-based and eye-gaze-based metrics to predict the population-wide behavior of moviegoers on new movie releases; as captured by the movie’s ticket sales. We evaluate the predictive power of the derived metrics and discuss, based on existing literature, their potential connection to the underlying cognitive processes that might drive moviegoers’ decision to watch a movie.

## Materials and Methods

### Experimental Paradigm

We record neural activity—through the use of EEG—and eye-gaze activity from a group of naive individuals while watching movie trailers of pre-selected movies for which the population-wide preference is captured by the film’s market performance (i.e., box-office ticket sales in the US).

#### Participants

A total of 27 participants (16 female, 11 male) were recruited for the study. All participants were recruited in Cyprus, were fluent in English and had self-reported normal or corrected-to-normal vision. The minimum, median and maximum ages of the participants were 19, 22 and 24, respectively. Participants were compensated for their participation in the study.

#### Video Stimuli

A database of 15 movie trailers, split into two stimuli sets, were used in this study. Movies were selected using a search of the boxofficemojo.com website. Specifically, we searched for movies in the action, adventure or thriller genre that premiered during the second or third quarter of 2014. The search focused only on the top 100 movies of each quarter, as ranked by their box office results. Movies that were released in less than 1000 theaters or had total revenue less than $10 million were excluded from the search results. This selection was made to ensure that only movies of reasonable quality and market reach were included in the final dataset. Movies that met the genre and inclusion criteria were ordered based on their box-office performance. To ensure maximum variability in commercial success, in the final database we included movies that came from both the highest and the lowest rankings of the resulting list. Official English trailers for the selected films were used in the study.

#### Data Collection Procedure

Data were collected in two different sessions to keep the duration of the experiment short and to minimize participant fatigue. Each of the sessions involved the presentation of a distinct set of movie trailers. Participants were randomly assigned to one of the sessions. Movie trailers were split into two stimuli sets; set A included eight of the fifteen trailers, and set B included the remaining seven. During the first session, 14 participants were exposed to movie trailers of set A, while in the second session 13 participants were presented with the movie trailers of set B.

At the onset of the testing, participants were seated in a comfortable chair and briefed on the objectives of the study. Participants were told that they would watch a set of movie trailers and that after each trailer they would have to answer a short questionnaire indicating the preferences about each trailer. Moreover, they were told that we would be collecting EEG and eye-tracking measures while watching those trailers. A quick preparation and calibration procedure (explained below), before the presentation of the movie trailers (7 or 8 trailers depending on the session) followed next. At the end of each trailer, an on-screen questionnaire was presented asking the participants to report the following: (a) the degree to which they liked the movie trailer; (b) whether they intended to watch the movie promoted by the movie trailer; and (c) whether they would share the movie trailers video on their Facebook account. After having watched all of the movie trailers once, the participants were shown the same trailers for the second time. The order of the movie trailers was randomized across participants, but the order was preserved for each participant in both presentations. The open source software OpenSesame (Mathôt et al., 2012) was used to present the trailers. The trailers were shown at a frame rate of 23 Hz and an aspect ratio of 4:3, with sound (through on monitor speakers). Screen resolution was set to 1024 × 758 for all movie trailers. The present study is carried out in accordance with the ethical standards and the study procedures were approved by the Cyprus Bioethics Committee, and a consent form was obtained from all participants prior to the experiment in accordance with the Declaration of Helsinki.

#### Behavioral Measures

Participants reported their preferences about the movie by completing an on-screen questionnaire, immediately after watching each trailer. Participants expressed their preferences for the film twice (once after the first viewing of the movie trailer and once after the second viewing). The questionnaire was composed of three questions. For the first question, participants indicated their liking of the film on a scale of 1–10 using a sequence of radio-buttons. For the second and third questions, the participants indicated their willingness-to-watch the film, and their willingness-to-refer the movie (i.e., to share the video on their Facebook account), by selecting either a yes or a no check box on the screen. Three behavioral metrics were constructed based on participants stated preferences. The *likeability metric (LM)* is defined as the average liking score across all participants and viewings of each movie-trailer. The *willingness-to-watch metric (WTW)* and the *willingness-to-refer metric* (WTR) were calculated as the fraction of yes-responses, across participants and viewings, to the willingness-to-watch and willingness-to-refer questions, respectively.

The reported liking scores of participants ranged between 0 and 9 (*M* = 4.6, SD = 2.4). There were no significant differences in reported liking scores between the first and second viewing for either collection sessions (first session: *F*_(1,179)_ = 0.02, *p* > 0.87 or second session *F*_(1,221)_ = 0.01, *p* > 0.93). Thus, the likability metric was calculated using the average participant responses from the two viewings.

#### Eye-Tracking Measures and Pre-Processing

During the experiment, eye-gaze data were collected at a sampling rate of 60 Hz and spatial accuracy below 0.5°. The eye-tracking unit was placed in front of the participant and below the stimulus presentation monitor, with the camera-to-eye-distance at about 60 cm. A 9-point calibration was executed before the experiment to ensure a correct mapping of the gaze data points and screen coordinates. Also, event markers were sent to the eye-gaze stream to allow synchronization of video frames and gaze data. The recorded eye-gaze-data stream was epoched between −2000 ms before each video start time and 2000 ms after the video finish time and then re-referenced to the video’s starting time. The Attentional-asynchrony metric (see “Statistical Analysis of the Prediction Metrics and Key Performance Indicators” section for details) was then calculated as a function of the epoched eye-gaze stream of all participants and viewings. The data analysis was performed using a custom Matlab code (Mathworks Inc., Natick, MA, USA; MATLAB, 2010).

#### EEG Measures and Pre-Processing

EEG data were collected using a BioSemi Active-two system (BioSemi, Amsterdam, Netherlands) at a sampling rate of 512 Hz. Participants were fitted with a standard 32-electrode cap following the international 10/20 system. The preparation procedure took about 10 min, during which time all electrodes were placed, and the DC offset of all sensors was kept below 20 μV. EEG data were collected for the entire duration of the experiment.

All EEG data preprocessing was performed offline using custom Matlab code (Mathworks Inc., Natick, MA, USA, MATLAB, 2010). As part of the preprocessing, the data were first downsampled to 256 Hz, and a software-based 1.5 Hz high-pass filter was employed on the continuous EEG signal, to remove DC drifts. Subsequently, 50 Hz and 100 Hz notch filters were applied to minimize the power-line noise interference, and all channels were then re-referenced to the average channel. The continuous EEG data was then epoched between 2000 ms prior the movie trailer’s start time and 2000 ms after its finish time and the baseline amplitude (i.e., from −2000 ms to 0 ms of the movie trailers onset) was removed from each epoch. Meta-data reflecting the exact timings of each epoch relative to the video-trailer timing were kept throughout the preprocessing procedure.

Following the preprocessing of the temporal signal, each epoch was transformed into the spectro-temporal domain by convolving the EEG signal of each epoch with a complex morlet kernel. The spectrotemporal coefficients were estimated for every half Hz between 1–80 Hz and resulted in a temporal resolution of 8 Hz. The instantaneous power of each spectrotemporal coefficient was calculated as the product of that coefficient with its complex conjugate. Thus, the EEG response of each participant on each movie-trailer was represented as the collection on instantaneous power measures at different channels, time and frequency combination. In our study, we considered the instantaneous power for the subset of frequency’s that corresponded to the beta and gamma bands. Specifically, we segmented the data into the following frequency bands 14–8 Hz, 16–18 Hz and 18–20 Hz for the beta band, and 40–48 Hz, 52–60 Hz, 52–70 Hz and 60–70 Hz for the gamma band.

### Attentional-Asynchrony and Cognitive-Congruency Metrics

#### Eye-Gaze Measurements and the Attentional-Asynchrony Metric

The calculation of Attentional-asynchrony metric was based on the Eye-Gaze Divergence Index introduced by Christoforou et al. (2015), which was shown to carry predictive information on the likability of narrative videos. Similar to the Eye-Gaze Divergence Index (Christoforou et al., 2015), Attentional- asynchrony was calculated as the proportion of the movie-trailer on which the visual attention of a group of viewer’s diverged. To quantify this proportion, the eye-gaze stream for each movie-trailer was first segmented into small overlapping windows, obtained in a time-resolved fashion by employing a sliding window with 250 ms duration and a shift of the window occurring every 50 ms (80% overlap between successive windows). Each window was subsequently classified as being either divergent or non-divergent; however, for the calculation of Attentional-asynchrony, a different classification criterion was used. Specifically, for each window, the set of pairwise Euclidian distances between all eye-gaze points of all participant was calculated, and a window was classified as being divergent if a fraction (in our study 30%) of the pair wise distances fell within a 90% confidence interval of the null-distribution. The null-distribution over pairwise distances was estimated by randomizing the order of windows, participants, and viewings for each movie trailer. Finally, the Attentional-asynchrony metric was calculated as the fraction of windows identified as being divergent.

#### EEG Measurements and Cognitive-Congruency Metric

Similarly, we used the epoched EEG measurement collected for each movie-trailer from all participants to define an aggregate metric of Cognitive-congruency. The objective of Cognitive-congruency metric was to identify and quantify coherence in the modulation patterns of instantaneous powers within the selected frequency bands. The rationale was that the presence of neuronal activity that was congruent across participants was an indication of the ability of the movie trailer to guide the viewer’s cognitive response consistently. We calculated the Cognitive-congruency of each movie trailer for different frequency bands (14–18 Hz, 16–18 Hz and 18–20 Hz covering the beta band and 40–48 Hz, 52–60 Hz, 52–70 Hz and 60–70 Hz covering the gamma band). This selection was motivated by recent results (Boksem and Smidts, 2015) that suggest the overall power in the beta and gamma bands might carry predictive information about the performance of movie trailers.

To calculate the Cognitive-congruency, we first identified a multivariate spatial component that maximizes the correlation of the instantaneous power of EEG measures between the first and second viewing of the movie-trailer across all participants. Specifically, we estimated a spatial component (projection) ***w*** ϵ ℝ^*D*^ such that ***w**^T^**R***_(1,2)_***w**/N(**w***) is maximized, where ***w*** is a weight vector, D corresponds to the number of EEG channels recorded, $\frac{1}{|S|.T}\;\sum_{s\in S}X_{\left(1,s\right)}X_{\left(2,s\right)}^{T}$ is the subject-aggregated covariance matrix of instantaneous powers, ***X***_*(i,s)*_ ϵ ℝ*^D × T^* corresponds to the epoched EEG trails (capturing instantaneous power in a particular frequency band) of the sth participant during the ith viewing of a movie-trailer, and *N**(w)** = **w**^T^(**R***_(1,1)_ + ***R***_(2,2)_)***w*** is a normalizing factor. The optimal ***w*** then refers to the solution to the generalized eigenvalue problem *λ*(***R***_(1,1)_ + ***R***_(2,2)_**)*w*** = ***R***_(1,2)_***w*** (Dmochowski et al., 2012). The Cognitive-congruency then corresponded to the eigenvalue which corresponded to the optimal projection vector ***w***. Moreover, with the optimal ***w***, we could recover its corresponding “forwards model” (Parra et al., 2008), which could be used to visualize the topographic distribution of maximally correlated EEG activity. All calculations for cognitive-congruency were implemented using custom Matlab code (Mathworks Inc.; MATLAB, 2010).

### Statistical Analysis of the Prediction Metrics and Key Performance Indicators

To evaluate the ability of Cognitive-congruency and Attentional-asynchrony to predict the commercial success of films, we used linear regression to model the relationship between these two metrics and a measure of box-office performance. We analyzed the relationship of between commercial success and each metric individually, and then we estimated a multiple regression model using both metrics.

#### Key Performance Indicators of Commercial Success

The commercial success of movies is typically measured regarding box-office sales. The film industry has long used a comprehensive system for measuring the post-air success of a movie; these measurements are collectively known as box-office performance and include the number of tickets sold and the total sales revenue from tickets on a daily, weekly and monthly basis. These metrics are of particular importance to the film industry as they provide a concrete framework for measuring changes in the overall success of the films.

In the present study, we used a key performance indicator (KPI) based on box-office measurements. This KPI is comprised of the movie’s recorded revenues during the opening weekend, divided by the total budget of the film to account for variability of marketing capacity and reach of different movies. Moreover, we used the same KPI for movie revenues for the first eight weekends after the movies’ release. All relevant data were obtained from the website boxofficemojo.com. Table 1 shows the titles of the movies used in the study along with their corresponding KPI score.

**Table 1:** Sales Performance key performance indicator (KPI) of each movie on Movie’s Premiere, and on subsequent weekends.

		WKND_j_ : J_th_ weekends after movie’s premiere							
Movie	Premiere	WKND_1_	WKND_2_	WKND_3_	WKND_4_	WKND_5_	WKND_6_	WKND_7_	WKND_8_
1	1.0975	0.4563	0.2372	0.1373	0.0866	0.0691	0.0489	0.0386	0.0184
2	0.3857	0.1907	0.1028	0.0717	0.0465	0.0342	0.0232	0.0180	0.0102
3	0.6737	0.2872	0.1867	0.0468	0.0086	0.0020	0.0006	0.0021	0.0007
4	0.5548	0.2478	0.1477	0.1012	0.1005	0.0609	0.0477	0.0308	0.0222
5	0.6207	0.3409	0.1764	0.0979	0.0505	0.0324	0.0166	0.0084	0.0051
6	0.5246	0.2282	0.1338	0.0953	0.0521	0.0388	0.0212	0.0116	0.0062
7	0.1034	0.0433	0.0159	0.0059	0.0013	0.0009	0.0007	0.0004	0.0003
8	0.3399	0.1318	0.0587	0.0181	0.0056	0.0007	0.0028	0.0020	0.0011
9	0.4271	0.2133	0.0986	0.0511	0.0255	0.0117	0.0063	0.0051	0.0031
10	0.2980	0.1101	0.0575	0.0213	0.0085	0.0064	0.0028	0.0021	0.0028
11	0.3469	0.1578	0.0761	0.0510	0.0293	0.0181	0.0066	0.0029	0.0045
12	0.3006	0.1514	0.0885	0.0589	0.0372	0.0137	0.0045	0.0018	0.0022
13	0.3938	0.1772	0.0577	0.0167	0.0086	0.0029	0.0013	0.0008	0.0026
14	0.2704	0.1203	0.0640	0.0274	0.0103	0.0045	0.0026	0.0015	0.0018
15	0.5529	0.1628	0.0758	0.0491	0.0308	0.0166	0.0095	0.0051	0.0036

*Key performance indicators (KPI) of each movie in the dataset used in the study. The ith row shows the sales performance KPI (revenue/budget) of the ith movie on the movie’s premiere weekend (column “Premiere”), and on the eight following weekends (columns WKND_1_,.. WKND_8_)*.

#### Statistical Models

In the first model, Attentional-asynchrony served as the independent variable and KPI during the premiere weekend of the movie served as the dependent variable. We explored the Attentional-asynchrony metric calculated on data from the first viewing and second viewing separately. Moreover, we examined the same model where the depended variable corresponded to the KPI of the movie during the first nine weekends.

In the second model, the Cognitive-congruency metric served as the independent variable and the sales performance KPI during the premiere weekend of the movie was the dependent variable. We explored the Cognitive-congruency metric calculated on beta and gamma frequency bands (see section on Cognitive-congruency); a separate univariate model was fitted for each of the selected instantiations of the Cognitive-congruency. As with Attentional-asynchrony, we also estimated a model where the depended variable corresponded to the sales performance KPIs of the movie during the first nine weekends. We report the explained variance of the model and regression statistics corrected for multiple comparisons using false discovery rate.

Finally, we considered a bivariate model where both Attentional-asynchrony and Cognitive-congruency served as independent variables and used the same dependent variable as in the univariate model. In the bivariate model, we only considered the Attentional-asynchrony from the first viewing of the trailer and Cognitive-congruency in the gamma band (60–70 Hz).

## Results

### Behavioral Results

Using the *LM* as an independent variable and the KPI during the premiere weekend as the dependent variable, the regression model showed that the LM was not a significant predictor of sales performance, *F*_(1,12)_ = 0.39, *R*^2^ = 0.02, *p* > 0.54). Similarly, in regression models where the *WTW* and WTR metric are the dependent variables, neither metric was a significant predictor of the sales performance KPI during the premiere weekend, WTW: *F*_(1,12)_ = 1.76, *R*^2^ = 0.11, *p* > 0.20, WTR: *F*_(1,12)_ = 1.75, *R*^2^ = 0.11, *p* > 0.20. Finally, correlation analysis showed a strong correlation among the three behavioral metrics, LM-WTW: *r* = 0.87, *p* < 0.001, LM-WTR: *r* = 0.67, *p* < 0.01, WTW-WTR: *r* = 0.79, *p* < 0.001. In all analyses, regression results are reported on data from 14 out of the 15 videos movie trailers. Trailer with vid:1 was removed as an outlier because its KPI score was four standard deviation above the mean KPI scores of the rest of the movies.

### Correlational Analysis Results

We first investigate the correlation between the sales performance KPI during the movie’s premiere and the proposed metrics. Correlation analysis showed a strong negative correlation between the attentional-asynchrony metrics and the sales performance KPI (Asy-viewing-1: *r* = −0.70, *p* < 0.01; Asy-viewing-2: *r* = −0.67, *p* < 0.01). The analysis also showed a strong positive correlation between the KPI and cognitive-congruency metrics, calculated on each of the four gamma band (*r* = 0.82–0.85, *p* < 0.001). A moderate negative correlation was observed between the KPI and the cognitive-congruency metric calculated on the beta range (16–18 Hz). However, it failed to reach significance (*r* = −0.45, *p* > 0.09). No correlation was established between the other two beta-band metrics.

### Attentional-Asynchrony Prediction Model Results

To investigate the capacity of Attentional-asynchrony metric to predict the sales performance KPI during the movie’s premiere, we employed two univariate regression models. In the first model, we considered the Attentional-asynchrony metric calculated on the first viewing of each movie trailer as the predictor, while in the second model we used Attentional-asynchrony calculated on data from the second viewing of the movie trailer. In both models, Attentional-asynchrony was regressed onto the sales performance KPI for the film’s premiere weekend. The results showed that Attentional-asynchrony was a significant predictor of sales performance KPI at the movie’s premiere. Specifically, Attentional-asynchrony calculated on eye-gaze data from the first viewing of the movie trailer predicted 49% of the model variance, *R*^2^ = 0.49, *F*_(1,12)_ = 11.53, *p* < 0.01, *R*^2^-adjusted = 0.44, *SE* = 0.14, Standard Error (SE) on *R*^2^ computer using bootstrap, while the corresponding metric calculated on eye-gaze data from the second viewing of the trailer predicted 44% of the variance, *R*^2^ = 0.44, *F*_(1,12)_ = 9.72, *p* < 0.01, *R*^2^-adjusted = 0.40, *SE* = 0.16. The attentional-asynchrony metrics for the two viewings were strongly correlated (*r* = 0.91, *p* < 0.001).

Subsequently, we investigated whether the capacity of Attentional-asynchrony (calculated on the first- and second- viewing independently) to predict the sales performance KPI during the movie premiere propagates to the sales performance KPIs of subsequent weeks. Table 2 shows the results of the mass-univariate regression where the Attentional-asynchrony acts as a predictor of the sales performance KPI of the movie for each one of the first 9 weeks.

**Table 2:** *R*^2^ scores of each of the the seven models when predicting sales performance KPI on each movie’s premiere and on subsequent weekends.

			WKND_j_ : J_th_ weekends after movie’s premiere							
Model	Premiere	WKND_1_	WKND_2_	WKND_3_	WKND_4_	WKND_5_	WKND_6_	WKND_7_	WKND_8_
Att-Asy-1	0.49* (0.14)	0.54** (0.11)	0.60** (0.10)	0.53** (0.15)	0.55** (0.25)	0.56** (0.25)	0.62** (0.25)	0.66** (0.22)	0.55** (0.25)
Att-Asy-2	0.44* (0.16)	0.43* (0.16)	0.51* (0.15)	0.30 (0.17)	0.25 (0.21)	0.29 (0.24)	0.34 (0.24)	0.37 (0.21)	0.27 (0.20)
Cogn-40-48	0.67* (0.09)	0.45* (0.13)	0.49* (0.15)	0.38 (0.18)	0.25 (0.16)	0.29 (0.17)	0.29 (0.16)	0.27 (0.16)	0.16 (0.13)
Cogn-52-60	0.52* (0.18)	0.31 (0.17)	0.34 (0.19)	0.34 (0.17)	0.28 (0.17)	0.30 (0.17)	0.31 (0.17)	0.28 (0.17)	0.18 (0.16)
Cogn-60-70	0.67** (0.11)	0.54* (0.14)	0.54* (0.15)	0.43 (0.17)	0.33 (0.18)	0.32 (0.19)	0.34 (0.18)	0.31 (0.18)	0.21 (0.17)
Cogn-52-70	0.72** (0.07)	0.55* (0.17)	0.54* (0.19)	0.49* (0.17)	0.40 (0.19)	0.35 (0.19)	0.36 (0.18)	0.34 (0.17)	0.24 (0.18)
Att+Cogn	0.73** (0.07)	0.63** (0.10)	0.66** (0.09)	0.59* (0.15)	0.57* (0.24)	0.57* (0.24)	0.63** (0.23)	0.66** (0.19)	0.56* (0.23)	

*The modulation of the R^2^ score for the seven prediction models. Single-starred R^2^ scores are significant at 0.05 threshold level, while doubled-starred R^2^ scores are significant at 0.01 threshold level. The significance is reported after correcting for multiple comparisons (using false-recovery-rate method). Within the parenthesis, below each R^2^ score, is the SE of R^2^ calculated using the bootstrap method. The model abbreviations are as follows: Att-Asy-1: Attentional-asynchrony metric during the first viewing is used as the independent variable; Att-Asy-2: Attentional-asynchrony metric during the first viewing is used as the independent variable; Cogn-X-Y: Cognitive-congruency metric calculated in the frequency range between X Hz and Y Hz is used as the predictor variable; Att+Cogn: the combined predictor model where both the Cognitive-congruency metric calculated on the frequency range 52–70 Hz and the Attentional-asynchrony metric (calculated on measurements from the first viewing) are used as predictor variables*.

### Cognitive-Congruency Prediction Model Results

First, we report results on the ability of the Cognitive-congruency EEG metric, calculated on gamma and beta bands, to predict sales performance KPI during the movie premiere. The univariate regression analysis shows that Cognitive-congruency calculated on each of the four gamma-band ranges significantly predicted the sales performance KPI during the premiere weekend. Specifically, Cognitive-congruency calculated in the gamma range 40–48 Hz was shown to predict 67% of the model variance, *R*^2^ = 0.67, *F*_(1,12)_ = 24.81, *p* < 0.001, *R*^2^-adjusted = 0.65, *SE* = 0.09), while the corresponding metric calculated on the 52–60 Hz range predicted 52% of the variance, *R*^2^ = 0.52, *F*_(1,12)_ = 13.20, *p* < 0.01, *R*^2^-adjusted = 0.48, *SE* = 0.18) and on the 60–70 Hz range predicted 67% of the variance, *R*^2^ = 0.67, *F*_(1,12)_ = 25.17, *p* < 0.001, *R*^2^-adjusted = 0.65, *SE* = 0.11). Moreover, regression analysis on Cognitive-congruency calculated in the broader gamma range (52–70 Hz) was found to explain 72% of the variance, *R*^2^ = 0.72, *F*_(1,12)_ = 31.45, *p* < 0.001, *R*^2^-adjusted = 0.70, *SE* = 0.07). On the other hand, Cognitive-congruency calculated on each of the two beta-band (14–16 Hz and 16–18 Hz) failed to predict the sales performance KPI during the movie premiere *R*^2^ = 0.01, *F*_(1,12)_ = 0.13*, ns*; *R*^2^ = 0.21, *F*_(1,12)_ = 3.28, *ns*, respectively).

**Figure 1:**
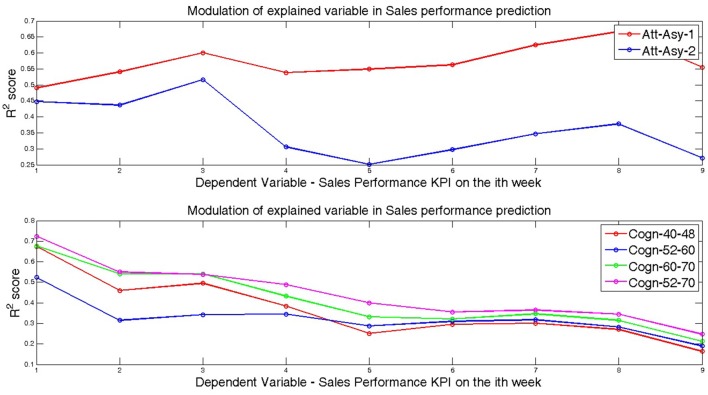
Shows the modulation of the *R*^2^ score for the Att-Asy-1, Att-Asy-2 models (top panel) and Cogn-40-48, Cogn-52-60, Cogn-60-70, Cogn-52-70 models (bottom panel) for the nine dependent variables (i.e., sales performance key performance indicator (KPI) on the movie premiere and the eight following weekends). The model abbreviations are as follows: Att-Asy-1: Attentional asynchrony metric during the first viewing is used as the independent variable; Cogn-52-70: Cognitive-congruency metric calculated in the frequency range between 52 Hz and 70 Hz is used as the predictor variable; Att+Cogn: The combined predictor model where both the Cognitive-congruency metric calculated on the frequency range 52–70 Hz and the Attentional-asynchrony metric (calculated on measurements from the first viewing) are used as predictor variables. The numerical values of *R*^2^ and Standard Error (SE) scores calculated using the bootstrap method are shown in Table 1.

Subsequently, we investigated if the capacity of Cognitive-congruency (calculated on the four gamma-band ranges) to predict the sales performance KPI during the premiere weekend also applies to subsequent weeks. Table 2 shows the results of the univariate regressions where the Cognitive-congruency acts as a predictor of the sales performance KPI of the movie for each of the first 9 weeks. The analysis showed that, indeed, Cognitive-congruency in the gamma-band continued to carry significant predictive power on sales performance KPI of the movie for up to 4 weeks, although the level of variance explained decrease every week. Figure 1 plots the *R*^2^ of explained variance under the model where Cognitive-congruency (for each of the four gamma-band ranges) served as a predictor, as a function of the nine subsequent weeks.

### Combined Eye-Tracking and EEG Prediction Model

**Figure 2:**
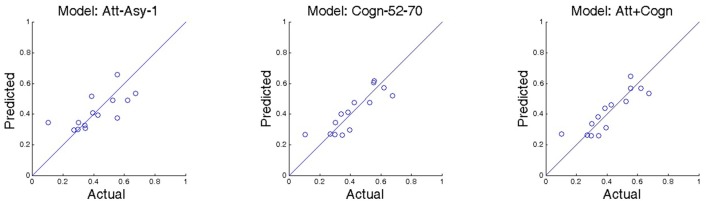
Shows scatter plots of actual vs. predicted sales performance KPI on the premiere of the movie for three different prediction models. The model abbreviations are as follows: Att-Asy-1: Attentional-asynchrony metric during the first viewing is used as the independent variable; Cogn-52-70: Cognitive-congruency metric calculated in the frequency range between 52 Hz and 70 Hz is used as the predictor variable; Att+Cogn: The combined predictor model where both the Cognitive-congruency metric (estimated on the frequency range 52–70 Hz) and the Attentional-asynchrony metric (calculated on measurements from the first viewing) are used as predictor variables.

**Figure 3:**
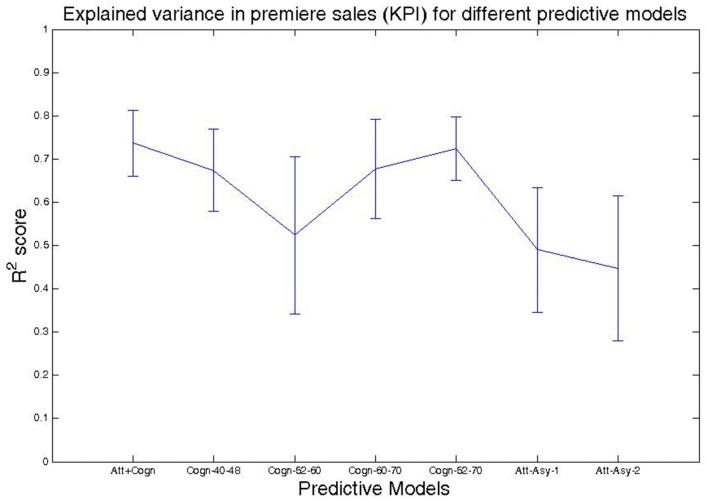
Shows the *R*^2^ score obtained by each of the seven models when predicting the sales performance (KPI) on a movie’s premiere. The error bars show the SE of *R*^2^ scores calculated using the bootstrap method.

**Figure 4:**
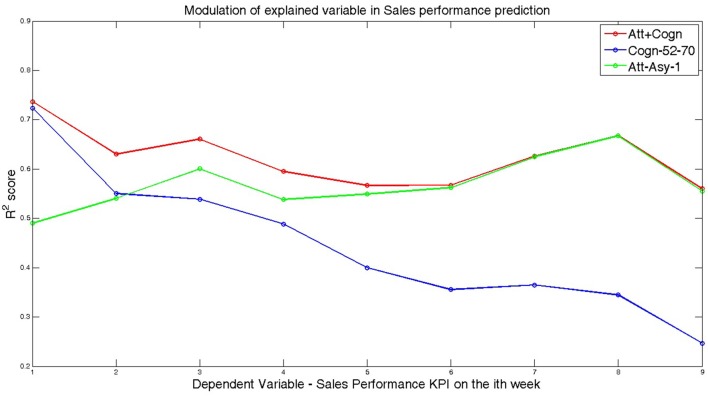
Shows the modulation of the *R*^2^ score for the Att-Asy-1, Cogn-52-70 and Att+Cogn models and the nine dependent variables (i.e., sales performance KPI on the movie premiere and the eight following weekends). The model abbreviations are as follows: Att-Asy-1: Attentional-asynchrony metric during the first viewing is used as the independent variable; Cogn-52-70: Cognitive-congruency metric calculated in the frequency range between 52 Hz and 70 Hz is used as the predictor variable; Att+Cogn: The combined predictor model where both the Cognitive-congruency metric calculated on the frequency range 52–70 Hz and the Attentional-asynchrony metric (calculated on measurements from the first viewing) are used as predictor variables.

**Figure 5:**
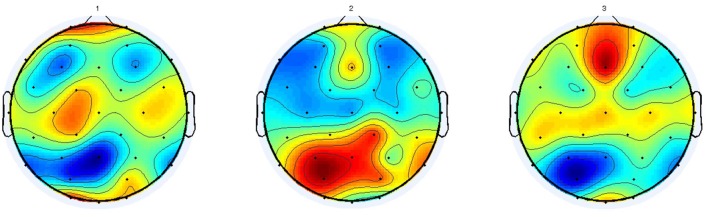
The average forward model of the spatial components used in calculating Cognitive-congruency scores.

Lastly, we consider the model where both Cognitive-congruency (calculated on the gamma band) and Attentional-asynchrony (computed on the first viewing of each movie trailer) serve as predictor variables of sales performance KPI for the film premiere weekend and subsequent weeks. The analysis shows that the combined model predicts 73% of the variance, *R*^2^ = 0.7370, *F*_(2,11)_ = 15.51, *p* < 0.001, *R*^2^-adjusted = 0.69, *SE* = 0.07, in sales performance KPI during the opening weekend. Subsequently, we investigated if the capacity of the combined model to predict the sales performance KPI during subsequent weeks. Table 2 shows the results of the regressions for the combined model for each one of the first 9 week. Figure 2 shows scattered plots of actual vs. predicted sales performance KPI on the movies premiere for three different prediction models. Figure 3 compares the of *R*^2^ score obtained by each of the seven models when predicting the sales performance (KPI) on a movie’s premiere. Figure 4 illustrates the modulation in explained variance across the 9 weeks for the Attentional-asynchrony model, the Cognitive-congruency model and the combined model, respectively. The forward model of the Cognitive-congruency model is illustrated in Figure 5.

## Discussion

In this study, we proposed a novel computational approach for extracting neurophysiological and eye-gaze based metrics to predict the population-wide behavior of movie goers. In particular, we derived two metrics, termed “Attentional-asynchrony”—calculated on eye-gaze data- and “Cognitive-congruency”—estimated on EEG data—and, evaluated the degree to which these metrics carry predictive information about the broader audience decisions to watch a movie. Towards that, we recorded neural activity—through the use of EEG—and eye-gaze activity from a group of naive individuals while watching movie trailers of pre-selected movies for which the population-wide preference is captured by the movie’s market performance (i.e., box-office ticket sales in the US). In this section, we discuss our key findings and the connection of the derived metrics to the underlying cognitive processes that might drive moviegoers’ decision to watch a movie.

A significant finding of this study is that neuroscience metrics obtained while people are observing movie trailers provides an alternative framework for predicting the overall performance of a film. Indeed, the results demonstrate that the proposed neuroscience-based metrics of Attentional-asynchrony and Cognitive-congruency can predict the commercial success of a given movie and explain a significant percentage of sales variability in box-office sales.

Specifically, when relating the Attentional-asynchrony metric to the commercial success of the films, we found that the metric carries significant predictive power. These results highlight the capacity of a movie trailer to guide viewers’ visual attention consistently as an important factor in predicting the success of a film. Moreover, because movie trailers are composed (primarily) of scenes from the actual movie, they act as indicators of the capacity of the movie itself to guide viewers’ attention in a consistent way. Further, the Attentional-asynchrony metric calculated on eye-gaze data obtained either during the first viewing or the second viewing exhibit equal predictive power and are highly correlated. This finding provides evidence for the robustness and consistency in the calculation of the metrics.

Intuitively, attentional-asynchrony quantifies the difficulty of a video stimuli to guide viewer’s visual attention consistently for the duration of the video. fMRI research corroborates the close link between eye-gaze and attention (Eckstein et al., 2017). Moreover, eye-gaze asynchrony measures have been associated with biological process relating to the allocation of attention (Christoforou et al., 2015). In this context, our finding might suggest that movie-trailers that are likely to influence the general population to decide to watch the film, are able to engage viewer’s visual in a consistent visual path, throughout the movie-trailer narrative.

We also tested the Cognitive-congruency metric, calculated on beta and gamma bands, as a predictor of the sales performance. Traditionally, researchers have related gamma-band activity to states of enhanced arousal and focused attention (Engel et al., 2001). Gamma-band synchronization between brain areas could reflect top-down control of attention modulated by the relevance of stimulus representation (Rodriguez et al., 1999). Moreover, gamma-band power was found to be enhanced during tasks involving object recognition (Rodriguez et al., 1999) and emotional evaluation tasks (Müller et al., 2000); both processes are most likely engaged during the viewing of movie clips (Boksem and Smidts, 2015). In this context, the proposed Cognitive-congruency metric—calculated on the gamma band—can be thought of as reflecting the capacity of the movie trailer to attract and maintain a viewer’s attention, and to engage an audience’s emotions. Thus, our finding suggests that the ability of the movie trailer to engage viewers, as quantified by the Cognitive-congruency metric, affects the likelihood that the general population will decide to watch the film, once exposed to movie-trailer.

Gamma activity is also strongly related to activation of the medial PFC (Mantini et al., 2007), an area in the brain which fMRI studies have found to be related to population-wide preferences and choices (Berns and Moore, 2012; Falk et al., 2012). Also, Howard et al. (2003) have reported that power in the gamma band is related to committing viewed materials to memory. Indeed, the medial PFC is strongly connected to the hippocampus (a brain area critically involved in memory formation) while gamma synchronization between the hippocampus and other brain regions are correlated with successful encoding of long-term memory (Fell et al., 2001). In this context, our findings suggest that gamma-modulation captured by the Cognitive-congruency metric reflects the capacity of the movie trailer to both capture and maintain viewer’s attention over time, and its ability to assist in memorability of the viewed materials. In turn, these characteristics of the movie trailer affect the likelihood that people will go and see the film.

When comparing the Cognitive-congruency metric (calculated on beta band power) to the population-wide commercial success of the movie, we found that the metric carried no predictive power. Previous research has linked medial frontal beta activity to reward processing including reward anticipation, rewards delivery, rewards evaluation and choice. Moreover, high-frequency oscillation (beta and gamma bands) are thought of being well suited to synchronize the different components of the reward network, as they allow for the communication and integration of information across distant brain areas. In fact, Boksem and Smidts (2015) have reported that medial frontal beta during viewing of a movie trailer relates to the individual’s preferences about the film. However, they note that beta activity carries no predictive information about the population-wide success of the film. Taken together, these findings might suggest that short-term rewards, revealed by higher beta activity, might reflect the immediate (short-term) evaluation of the movie. However, the effect of the rewards is temporary and does not affect the likelihood of viewers to go and watch the film.

Putting all the above together, the findings suggest that movie-trailers are more successful in influencing viewer’s to watch a movie when they capture and retain viewer’s attention and potentially engage viewer’s emotion; as attentional-asynchrony and cognitive-congruency metrics measure those. The most successful movie-trailers though, also trigger the long-term memory encoding process in the brain, as the gamma-band cognitive-congruency metrics capture that, and thus facilitate memorization of the film’s narrative. On the other hand, movie-trailers that engage the reward system of viewer’s brain, as captured by the beta-band cognitive-congruency metric, do not necessarily translate to increase in the likelihood of viewers to go and watch the film.

Finally, we explored a combined model where both Attentional-asynchrony and Cognitive-congruency metrics are used as predictors of population-wide success of the movies. The findings suggest that the two predictors provide complementary predictive information and likely capture different factors affecting the population’s decision to see a movie. The combined model explained significantly higher variability in sales performance of the movie for the first three weekends after the film’s premiere, while it explained at least as much variability as either of the individual models on the remaining weekend sales. Interestingly, by inspecting the modulation of explained variance under the different models (i.e., Att-Asy-1 and Cogn-52-70) across consecutive weekend sales performance, we found that Cognitive-congruency was a significantly stronger predictor of sales performance during the movie opening weekend. In turn, Attentional-asynchrony was a more reliable predictor of the sales performance on the weekends after the premiere. These findings provide additional evidence on the complementarity of predictive information from the two metrics. On the one hand, these results suggest that Cognitive-congruency, and thus the capacity of the trailer to both capture and maintain viewer’s attention over time, functions as a robust metric for the decision made by frequent moviegoers about the quality of a movie. Our results also suggest that this applies in particular during the movie’s opening weekend when the movie is usually attended by regular movie–goers and fans of the film. On the other hand, the decisions of infrequent or casual moviegoers, which are likely to attend the film in following weekends after the movie’s opening weekend, are better characterized by Attentional-asynchrony metric. From a practical point of view, given that the opening weekend accounts for 30%–50% of the total sales within the first nine weekends of a movie’s release, the power of the Cognitive-congruency metric to predict sales during the film’s opening weekend remains particularly important from a financial point of view.

Finally, the results showed a high correlation between the three behavioral measures of likeability, WTW and WTR metrics. However, none of the traditional behavioral measures were found to be significant predictors of population-wide commercial success of the movies. That the stated preference-based measures did not predict commercial success could be attributed to the sample size of the present study. Indeed, our findings are consistent with the work of Boksem and Smidts (2015), in which behavioral measures obtained on a small sample size (comparable to the current study) fail to predict broad population preferences. Current industry practices that rely on traditional behavioral measures require much larger sample sizes (>400 respondents) than the sample size used in this study. Our result could be seen as evidence of the reasonable variability that exists among stated preference measures. In contrast, the neuronal based metrics proposed in this article were found to extract predictive information of commercial success on different movies from a small sample size, suggesting that neural based metrics better reflect more accurate measures of the effect of a movie trailer on viewers.

Like previous research in neuromarketing research, the present study exhibits the limitations of correlational studies. For example, in the present study, we recorded participants’ likeness of a movie, but we did not use this data to screen participants or movies. Our approach was based on the assumption that most of what we think we know is what we have been conditioned to know (Das, 2016). Nevertheless, future studies should control for the possible effects of movie likeness. Also, a future direction of the work in this area is to implement a broad range of criteria for movie selection that relate to the production technique of the movies (e.g., loudness, montage, sound effects), apart from those used in the present study. Another future research direction would be to study the relationship of the proposed metrics to pertinent factors of a movie’s sales performance, such as film familiarity, the popularity of the actors and cultural familiarity (Hennig-Thurau et al., 2007). Factoring into the equation such features will help to examine further not only the direction but also the causality of the relations identified in this and other studies.

In summary, in this article, we propose a new computational approach for extracting a neural-based and an eye-gaze-based metrics and investigated their capacity to provide valuable and significant insights in predicting the population-wide behavior of movie goers. The first metric termed “Attentional-asynchrony” relies on eye-gaze data while the second metric termed “Cognitive-congruency” is estimated on selected frequency bands from the raw-EEG data. We provide evidence that such metrics provide significant and valuable insights in predicting sales performance of the movie during premiere as well as subsequent weekends, thus anticipating the commercial success of each film. Moreover, we discuss, in the context of existing literature, the possible relations of the derived neural metrics to cognitive states of enhanced arousal and focused attention, the encoding of long-term memory and the synchronization of different areas of the brain’s rewards network. The proposed approach can be employed to pre-test a movie-trailer and anticipate the commercial success of the movie or TV series, thereby helping to inform the marketing strategy of the film before its release. Moreover, the proposed neurophysiological and eye-gaze based metrics could be used as markers in cinematics studies to help investigate the impact of movie features and filming techniques (scene transition/animations/special effect) to moviegoers’ behavior. Finally, beyond the practical implication in predicting and understanding the behavior of moviegoers, the proposed approach can facilitate the use of video stimuli in neuroscience research; such as the study of individual differences in attention-deficit disorders, and the study of desensitization to media violence.

## Author Contributions

CC and MT developed the study concept, designed and performed the experiments. CC analyzed the data and wrote the article. MT and TCP edited the manuscript. FC critically reviewed and evaluated the manuscript. All authors discussed the results and implications and commented on the manuscript at all stages.

## Conflict of Interest Statement

The corresponding author served as CTO at R.K.I Leaders Ltd. The other authors declare that the research was conducted in the absence of any commercial or financial relationships that could be construed as a potential conflict of interest.

## Footnotes

^1^Even though the underlying measurements (i.e., raw EEG) tend to be noisy, the extracted metric/features’ noise across participants are expected to be relatively small.
